# The role of probiotics and synbiotics on treatment of gestational diabetes: systematic review and meta-analysis

**DOI:** 10.1016/j.xagr.2023.100285

**Published:** 2023-10-26

**Authors:** Arresta Vitasatria Suastika, I Gde Raka Widiana, Ni Nengah Dwi Fatmawati, Ketut Suastika, Ivana Beatrice Paulus, I Nengah Sujaya

**Affiliations:** 1 Udayana Medical School, Denpasar, Bali, Indonesia (Dr A Suastika); 2 Faculty of Medicine, Department of Microbiology, Udayana University, Bali, Indonesia (Dr N Fatmawati); 3 Department of Internal Medicine, Udayana University, Bali, Indonesia (Drs I Widiana and K Suastika); 4 Wangaya General Hospital, Denpasar, Bali, Indonesia (Dr Paulus); 5 Faculty of Medicine, School of Public Health, Udayana University, Bali, Indonesia (N Sujaya PhD)

**Keywords:** gestational diabetes mellitus, probiotic, synbiotics

## Abstract

**OBJECTIVE:**

This review investigated the efficacy of probiotics and/or synbiotics in gestational diabetes mellitus treatment by targeting insulin resistance, lipid metabolism, and anti-inflammatory effects in an updated trial.

**DATA SOURCES:**

The literature review was performed using the key words “Probiotics,” “Synbiotics,” and “Gestational Diabetes” in several databases, including PubMed, ScienceDirect, and the Cochrane Central Register of Controlled Trials.

**STUDY ELIGIBILITY CRITERIA:**

Eligible publication was screened independently by 2 reviewers. Studies included provided at least 1 of the following outcomes: (1) blood glucose marker, including fasting blood glucose level, fasting serum insulin level, and homeostasis model assessment insulin resistance; (2) blood lipid profiles, including triglycerides, low-density lipoprotein cholesterol, and high-density lipoprotein cholesterol; and (3) nitric oxide and C-reactive protein.

**METHODS:**

All studies were reviewed using the critical appraisal Cochrane risk-of-bias tool for randomized trials. The descriptions of the extracted data were guided by the Preferred Reporting Items for Systematic Reviews 2020 statement with the Grading of Recommendations Assessment, Development, and Evaluation approach. This study was registered on the International Prospective Register of Systematic Reviews database (identification number: CRD42022375665).

**RESULTS:**

From 13 randomized controlled trials involving 896 patients, individuals with probiotic had significant reduction on homeostasis model assessment insulin resistance (mean difference, −0.72; 95% confidence interval, −1.07 to −0.38; *I^2^*, 96%; *P*=.00), fasting blood glucose level (mean difference, −3.79; 95% confidence interval, −6.24 to −1.34; *I^2^*, 93%; *P*=.00), and insulin level (mean difference, −2.43 mg/dL; 95% confidence interval, −3.37 to −1.48; *I^2^*, 54%; *P*=.00). Meanwhile for profile lipid, significant reduction of the mean difference was observed in the triglyceride (mean difference, −17.73 mg/dL; 95% confidence interval, −29.55 to − 5.9; *P*=.003) and C-reactive protein (mean difference, −1.93 dL; 95% confidence interval, −2.3 to −1.56; *P*=.00).

**CONCLUSION:**

Probiotic and synbiotic supplementations reduced the risk of insulin resistance and improved glycemic control, blood lipid profiles, and inflammation in women with gestational diabetes mellitus. Probiotics may be a viable option for gestational diabetes mellitus treatment; however, large-scale, well-designed randomized controlled trials with longer follow-up periods are required before they can be recommended to patients.

**AJOG Global Reports at a Glance**

**Why was this study conducted?**

The metabolic effects of probiotics in women with gestational diabetes mellitus (GDM) remain unclear.

**Key findings**

From 13 randomized controlled trials involving 896 patients, individuals with probiotics had significant reduction on homeostasis model assessment insulin resistance (mean difference [MD], −0.72; 95% confidence interval [CI], −1.07 to −0.38; *I^2^*, 96%; *P*=.00), fasting blood glucose level (MD, −3.79; 95% CI, −6.24 to −1.34; *I^2^*, 93%; *P*=.00), and insulin level (MD, −2.43 mg/dL; 95% CI, −3.37 to −1.48; *I^2^*, 54%; *P*=.00). The triglyceride (MD, −17.73 mg/dL; 95% CI, −29.55 to −5.90; *P*=.003) and C-reactive protein (MD, −1.93 dL; 95% CI, −2.30 to −1.56; *P*=.00) levels differed significantly.

**What does this add to what is known?**

Probiotic and synbiotic supplementations reduce the risk of insulin resistance and improve glycemic control, blood lipid profile, and inflammation in women with GDM.

## Introduction

Gestational diabetes mellitus (GDM) is defined as intermediate hyperglycemia, including impaired glucose tolerance and impaired fasting glycemia, diagnosed at any time during pregnancy.^1^ The prevalence of GDM in Southeast Asian countries (median, 15.0%) ranks second to Middle Eastern countries and some North African countries, with a median of 15.2%.^2^ Epidemiologic studies have identified several risk factors for GDM, such as advanced maternal age, ethnicity, overweight or obesity, multiple pregnancies, previous history of GDM, and family history of type 2 diabetes mellitus (T2DM).^3^ Diabetes mellitus during pregnancy is associated with adverse maternal and perinatal outcomes,^4^ including short-term (eg, preeclampsia, hypoglycemia, excessive adiposity, shoulder dystocia, miscarriage, preterm birth, and macrosomia) and long-term (eg, maternal and child obesity and development of T2DM) morbidities.^5^^,^^6^ Women with hyperglycemia detected during pregnancy are at greater risk of adverse pregnancy outcomes, notably macrosomia and preeclampsia, even after excluding the more severe cases of hyperglycemia that require treatment. The treatment of GDM is effective in reducing macrosomia, large for gestational age, shoulder dystocia, and preeclampsia or hypertensive disorders during pregnancy.^1^

Probiotics were defined by the Food and Agriculture Organization and the World Health Organization as “live microorganisms [that], when administered in adequate amounts, confer a health benefit on the host.”^7^ Prebiotics are generally defined as nondigestible food ingredients, including fructooligosaccharides and inulin, which beneficially affect the host by selectively stimulating the growth and/or activity of one or a limited number of bacterial species in the colon.^8^ Meanwhile, synbiotics are a mixture of probiotics and prebiotics that beneficially affect the host by improving the survival and implantation of live microbial dietary supplements in the gastrointestinal tract of the host.^9^ Probiotic or synbiotic based supplements have been found to improve the outcomes of patients with GDM concerning the markers of glucose and lipid metabolism, markers of inflammatory and oxidative stress, pregnancy, and newborn outcomes.^10^^,^^11^ Prebiotics enhance the beneficial effect of probiotics by stimulating their growth, activity, or both.^12^ However, the effect of prebiotics alone on diabetes mellitus did not show statistical significance. Although some studies involving rodent models have reported positive effects, human studies are still lacking, particularly among those with GDM.^13^^,^^14^ Probiotics combined with prebiotics are termed synbiotics; these were included in this study to assess the total effect required to improve the viability of the probiotics.^15^ However, some clinical randomized controlled trials (RCTs) have reported no significant difference in insulin resistance^16^ or pregnancy outcomes^17^ between the probiotic and synbiotic and placebo groups.

## Objectives

Considering these inconsistent effects, this systematic review aimed to evaluate the efficacy of probiotics and/or synbiotics in GDM treatment, targeting insulin resistance, lipid metabolism, anti-inflammatory aspects, and pregnancy outcomes.

## Methods

This meta-analysis was performed following the Preferred Reporting Items for Systematic Reviews (PRISMA) 2020 guidelines. The study was registered on the International Prospective Register of Systematic Reviews database (identification number: CRD42022375665).

### Criteria, information sources, and search strategy

Literature review searching was performed using the following key words: “Probiotics,” “Synbiotics,” and “Gestational Diabetes.” We searched the literature in the following databases: PubMed, ScienceDirect, and the Cochrane Central Register of Controlled Trials. Eligible publications were screened independently by at least 2 reviewers who provided one of the following outcomes: (1) blood glucose markers, including fasting blood glucose (FBG) level, fasting serum insulin (FSI) level, and homeostasis model assessment insulin resistance (HOMA-IR), and (2) blood lipid profiles, including triglyceride (TG), low-density lipoprotein (LDL) cholesterol, and high-density lipoprotein (HDL) cholesterol; and (3) nitric oxide (NO) and C-reactive protein (CRP).

### Study selection process

The inclusion criteria were as follows: (1) diagnosis of GDM, (2) written in English, (3) published after 2013, and (4) a clinical trial design. Letters, viewpoints, and reviews were excluded from the analysis. Previous studies have focused on the role of probiotics in the treatment of GDM.

### Data extraction

Any controversies were resolved through discussions. The eligible studies were exported and extracted according to the PRISMA statement and checklist. Grading of Recommendations Assessment, Development, and Evaluation (GRADE) criteria was used to analyze and report the level of evidence.^18^

### Risk of bias of the included studies

All studies were assessed and scored using the critical assessment tool checklist of the Cochrane risk-of-bias tool for randomized trials. Each reviewer independently assessed the titles and abstracts to select potentially eligible studies whose full texts fulfilled the inclusion and exclusion criteria. The authors screened the manuscripts to identify articles related to the outcomes and excluded duplicates. Non-English articles, non–full-text studies, nonhuman trials, trials with insufficient data, and studies in which the intervention groups applied other lifestyle interventions were also excluded. A third reviewer resolved any disagreements between the 2 reviewers. The reference lists of the eligible studies and relevant reviews were manually checked for additional studies. Author, year of publication, sampling period, study location, number of participants, mean age, weight of participants, diagnostic criteria for GDM, details of intervention (probiotic strains, counts, and duration), and main outcome were all collected from eligible studies. Each author independently evaluated and assessed the risk of bias in all included studies and completed the Cochrane Handbook for RCTs.^19^

### Data synthesis and analysis

The software Review Manager (version 5.4.1; Cochrane Collaboration, Copenhagen, Denmark), OpenMetaAnalys (Brown University, Providence, RI), and Stata (version 17; StataCorp LLC, College Station, TX) were used to examine the effect of probiotics and synbiotics on the glycemic, inflammatory, and lipid levels of the patients. The mean difference (MD) was used to present the effects, and the Mantel-Haenszel formula was used to calculate continuous variables. Dichotomous variables were reported with 95% confidence intervals (CI) for the MD. Of note, 2-tailed *P* values were used, and *P* values of <.05 were considered statistically significant, with a random-effect model. Heterogeneity was evaluated using the Q-statistic test, and the *I^2^* test included calculation variations in each study because of clinical or methodological heterogeneity.^19^ Regression and sensitivity analyses were used to determine the bias. Leave-out analysis was used in this study, along with sensitivity analysis by removing studies 1 by 1 to see the effects provided in the results.

## Results

### Study selection

**Figure 1:**
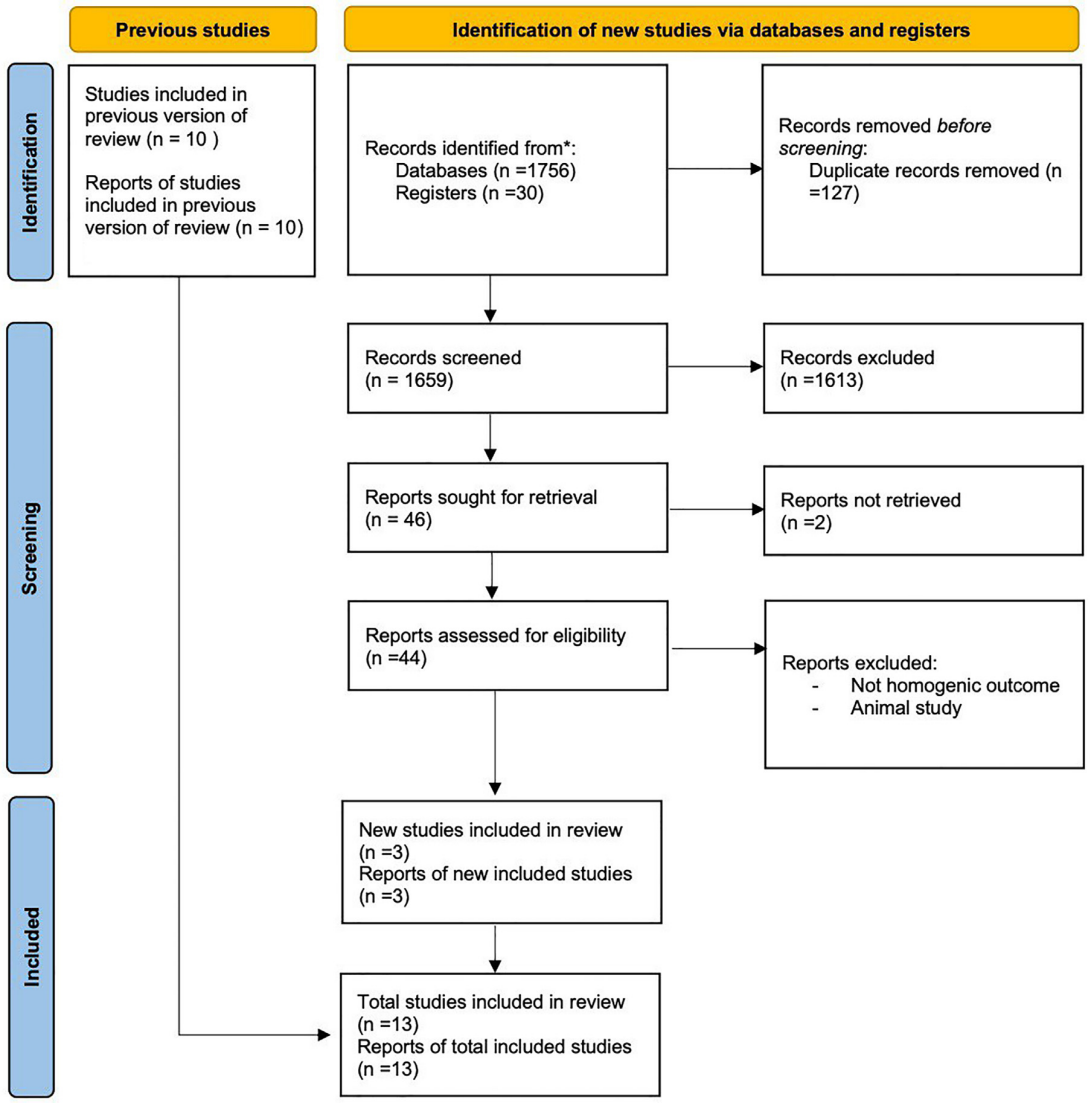
Flowchart diagram of PRISMA *PRISMA*, Preferred Reporting Items for Systematic Reviews.

The literature search and study selection processes are shown in Figure 1. Overall, 1786 citations were collected from the initial literature search of several databases. After excluding duplicates and articles based on title and abstract review, 44 potentially eligible studies were included in the full-text review. Ultimately, 13 studies that reported the effects of probiotics or synbiotics on GDM were included in our review. The selection process algorithm is illustrated in Figure 1 and Table 1.

Suastika. The role of probiotics and synbiotics on treatment of gestational diabetes. Am J Obstet Gynecol Glob Rep 2023.

**Table 1:** Methodology of search

No	Terminology	Hits
PubMed	(“diabetes, gestational”[MeSH Terms] OR (“diabetes”[All Fields] AND “gestational”[All Fields]) OR “gestational diabetes”[All Fields] OR (“gestational”[All Fields] AND “diabetes”[All Fields] AND “mellitus”[All Fields]) OR “gestational diabetes mellitus”[All Fields]) AND (“synbiote”[All Fields] OR “synbiotes”[All Fields] OR “synbiotic”[All Fields] OR “synbiotically”[All Fields] OR “synbiotics”[All Fields] OR (“probiotic s”[All Fields] OR “probiotical”[All Fields] OR “probiotics”[MeSH Terms] OR “probiotics”[All Fields] OR “probiotic”[All Fields])) Translations gestational diabetes mellitus: “diabetes, gestational”[MeSH Terms] OR (“diabetes”[All Fields] AND “gestational”[All Fields]) OR “gestational diabetes”[All Fields] OR (“gestational”[All Fields] AND “diabetes”[All Fields] AND “mellitus”[All Fields]) OR “gestational diabetes mellitus”[All Fields] synbiotic: “synbiote”[All Fields] OR “synbiotes”[All Fields] OR “synbiotic”[All Fields] OR “synbiotically”[All Fields] OR “synbiotics”[All Fields] probiotic: “probiotic's”[All Fields] OR “probiotical”[All Fields] OR “probiotics”[MeSH Terms] OR “probiotics”[All Fields] OR “probiotic”[All Fields]	158
CENTRAL	(((Gestational Diabetes) OR (Gestational Diabetes Mellitus)) AND ((probiotic") OR (Synbiotic")))	30
ScienceDirect	(((Gestational Diabetes) OR (Gestational Diabetes Mellitus)) AND ((probiotic") OR (Synbiotic")))	1593

*CENTRAL*, Cochrane Central Register of Controlled Trials; *MeSH*, Medical Subject Headings.

### Characteristics of included studies

**Figure 2:**
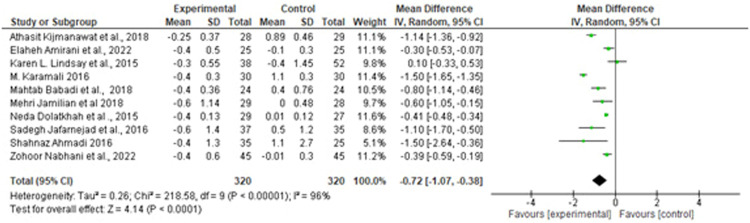
Forest plot mean difference of HOMA-IR *CI*, confidence interval; *HOMA-IR*, homeostasis model assessment insulin resistance; *IV*, inverse variance; *SD*, standard deviation.

**Figure 3:**
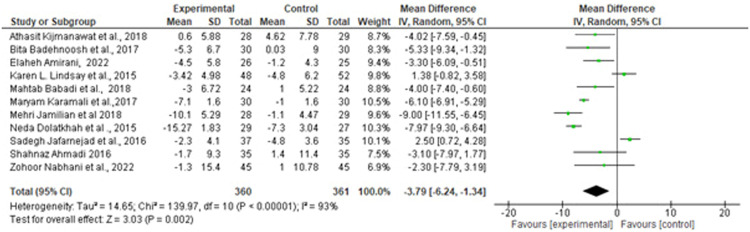
Forest plot mean difference of FBG (mmol/L) *CI*, confidence interval; *FBG*, fasting blood glucose; *IV*, inverse variance; *SD*, standard deviation.

**Figure 4:**
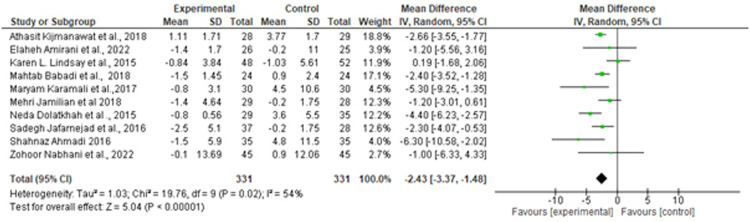
Forest plot mean difference of insulin (mmol/L) *CI*, confidence interval; *IV*, inverse variance; *SD*, standard deviation.

**Figure 5:**
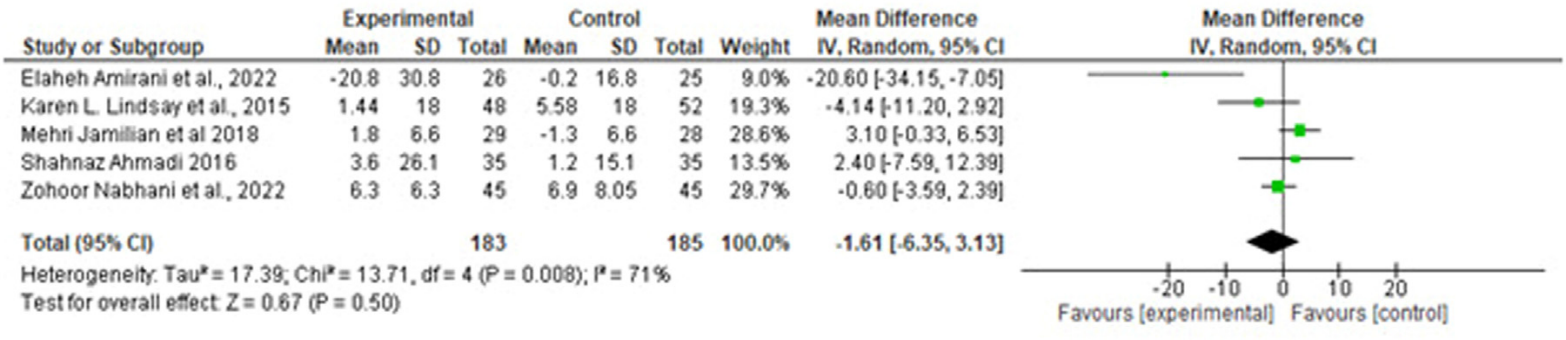
Forest plot mean difference of low-density lipid (mg/dL) *CI*, confidence interval; *IV*, inverse variance; *SD*, standard deviation.

**Figure 6:**
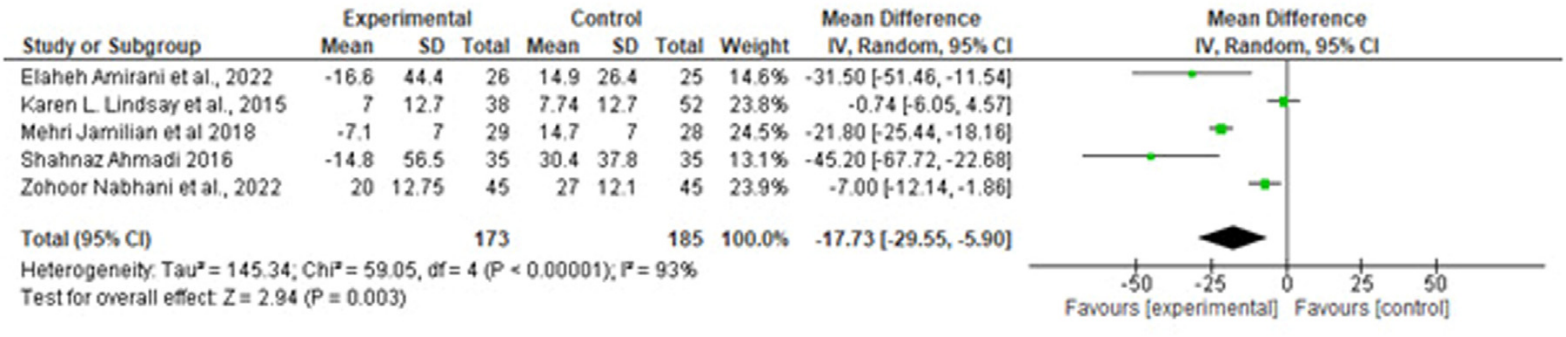
Forest plot mean difference of triglyceride (mg/dL) *CI*, confidence interval; *IV*, inverse variance; *SD*, standard deviation.

**Figure 7:**
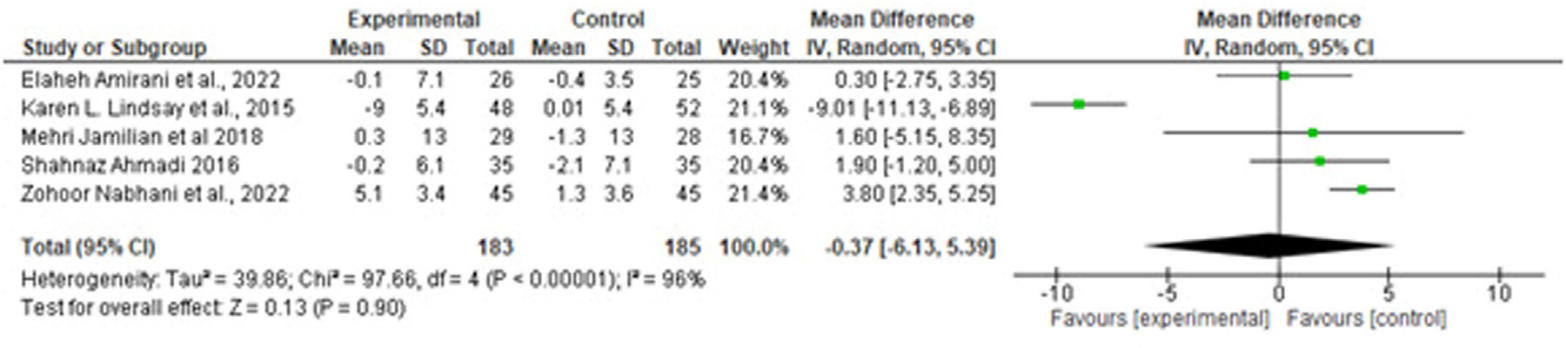
Forest plot mean difference of high-density lipid (mg/dL) *CI*, confidence interval; *IV*, inverse variance; *SD*, standard deviation.

Table 1 provides a detailed overview of the baseline characteristics of the included studies. Of these, 10 trials were on probiotics, and 3 trials were on synbiotic. Moreover, 13 studies administered at least 2 bacterial species, whereas the remaining studies used a combination of more than 2 strains. The composition of probiotics varied across the studies; however, all trials included *Lactobacillus*, except 2 that used *Bifidobacterium.* In addition, selenium and vitamin D were added to the flora of probiotic yogurt in the 2 study groups. The mean age of the participants ranged from 26.2 to 33.5 years, and the duration of the intervention ranged from 4 to 8 weeks. Furthermore, 11 of 12 articles were from Iran, and the remaining articles were from Thailand. The results of the meta-analyses are described in Figure 1, Figure 2, Figure 3, Figure 4, Figure 5, Figure 6, Figure 7.

Suastika. The role of probiotics and synbiotics on treatment of gestational diabetes. Am J Obstet Gynecol Glob Rep 2023.

Suastika. The role of probiotics and synbiotics on treatment of gestational diabetes. Am J Obstet Gynecol Glob Rep 2023.

Suastika. The role of probiotics and synbiotics on treatment of gestational diabetes. Am J Obstet Gynecol Glob Rep 2023.

Suastika. The role of probiotics and synbiotics on treatment of gestational diabetes. Am J Obstet Gynecol Glob Rep 2023.

Suastika. The role of probiotics and synbiotics on treatment of gestational diabetes. Am J Obstet Gynecol Glob Rep 2023.

Suastika. The role of probiotics and synbiotics on treatment of gestational diabetes. Am J Obstet Gynecol Glob Rep 2023.

### Risk of bias of included studies

Table 2 presents crucial appraisals of the eligible studies. We used GRADE to assess the level of evidence, which was found to be moderate to high (Table 3). The quality of the studies was generally good, with most being double-blind studies with concealment.

**Table 2:** Overview of eligible studies

Author	Number and country of participants	Age	Gestational age before the intervention	Diagnostic criteria	Intervention	Duration	Total dose (CFU)	Outcome
Kijmanawat et al,^20^ 2019	Thailand	32.50±5.02 vs 30.72±5.05	27.29±2.42 vs 27.97±2.54	2-h OGTT at 24–28 wk of gestation	*Bifidobacterium bifidum* and *Lactobacillus acidophilus* vs a placebo (28/29)	4 wk	2 × 10^9^	Metabolic parameters
Badehnoosh et al,^17^ 2018	Iran	27.80±3.70 vs 28.80±5.40	25.60±1.20 vs 25.70±1.00	2-h OGTT at 24–28 wk of gestation	*Lactobacillus casei* and *B bifidum* vs placebo (30/30)	6 wk	2 × 10^9^	Inflammatory marker, oxidative stress, and pregnancy outcome
Jafarnejad et al,^21^ 2016	Iran	32.40±3.10 vs 31.90±4.00	26.40 vs 26.60	2-h OGTT	*Streptococcus thermophilus, Bifidobacterium breve, Bifidobacterium longum, Bifidobacterium infantis, L acidophilus, Lactobacillus plantarum, Lactobacillus paracasei*, and *Lactobacillus delbrueckii* subsp *Bulgaricus* vs placebo (41/41)	8 wk	112.5 × 10^9^	Metabolic parameters
Lindsay et al,^22^ 2015	Iran	33.00±4.80 vs 33.50±5.00	29.60±2.50 vs 29.80±2.50	IGT or GDM (3-h OGTT)	*Lactobacillus salivarius* UCC118 vs placebo (74/75)	6 wk	1 × 10^9^	Metabolic parameters and pregnancy outcome
Karamali et al,^23^ 2016	Iran	29.70±4.00 vs 31.80±6.00	NR	2-h OGTT at 24–28 wk of gestation	*L acidophilus, L casei*, and *B. bifidum* vs placebo (30/30)	6 wk	6 × 10^9^	Metabolic parameters
Karamali et al,^11^ 2018	Iran	27.20±5.90 vs 26.20±3.10	NR	2-h OGTT at 24–28 wk of gestation	*L acidophilus* strain T16 (IBRCM10785), *L casei* strain T2 (IBRC-M10783), and *B bifidum* strain T1 (IBRC-M10771) plus 800 mg inulin vs placebo (30/30)	6 wk	8 × 10^9^	Inflammatory marker and pregnancy outcome
Babadi et al,^10^ 2019	Iran	29.00±4.20 vs 28.80±4.30	NR	2-h OGTT at 24–28 wk of gestation	*L acidophilus, L casei, B bifidum*, and *Lactobacillus fermentum* vs placebo (24/24)	6 wk	8 × 10^9^	Inflammatory marker, oxidative stress, and metabolic parameter
Nabhani et al,^16^ 2018	Iran	30.30±5.60 vs 29.40±5.80	26.20±2.30 vs 25.80±1.80	2-h OGTT at 24–28 wk of gestation	*L acidophilus, L plantarum, L fermentum, Lactobacillus gasseri*, and 38.5 mg of fructooligosaccharides vs placebo (45/45)	6 wk	77.5 × 10^9^	Inflammatory marker, oxidative stress, and metabolic parameter
Hajifaraji et al,^24^ 2018	Iran	28.10±6.25 vs 26.50±5.24	NR	2-h OGTT at 24–28 wk of gestation	*L acidophilus* LA-5, *Bifidobacterium* BB-12, *Streptococcus thermophilus*, and *L delbrueckii bulgaricus* vs placebo (29/27)	8 wk	>4 × 10^9^	Inflammatory marker and oxidative stress
Dolatkhah et al,^25^ 2015	Iran	27.30±5.80	28.14±6.24 vs 28.14±6.24	2-h OGTT at 24–28 wk of gestation	*L acidophilus* LA-5, *Bifidobacterium* BB-12, *S thermophilus* STY-31, and *L delbrueckii bulgaricus* (29/27)	6 wk	>4 × 10^9^	Metabolic parameter
Ahmadi et al,^26^ 2016	Iran	28.50±5.80 vs 287.00±3.40	NR	2-h OGTT at 24–28 wk of gestation	*L acidophilus, L casei*, and *B bifidum* with inulin vs placebo (35/35)	6 wk	8 × 10^9^	Metabolic parameter
Jamilian et al,^27^ 2019	Iran	31.20±5.90 vs 29.90±3.70	NR	2-h OGTT at 24–28 wk of gestation	*L acidophilus, B bifidum, L reuteri*, and *L fermentum* vs placebo (29/28)	6 wk	8 × 10^9^	Inflammatory marker, oxidative stress, and pregnancy outcome
Amirani et al,^28^ 2022	Iran	28.60±3.80 vs 27.10±5.80	NR	1-h and 2-h OGTT at 24–28 wk of gestation	*L acidophilus, B bifidum, B lactis*, and *B longum* plus selenium vs placebo (26/25)	6 wk	8 × 10^9^	Metabolic parameter

*IGT*, impaired glucose tolerance; *NR*, Not reported; *OGTT*, oral glucose tolerance test.

**Table 3:** GRADE approach

Certainty assessment							No. of patients		Effect		Certainty	Importance
No. of studies	Study design	Risk of bias	Inconsistency	Indirectness	Imprecision	Other considerations	Intervention	Comparison	Relative risk (95% CI)	Absolute risk (95% CI)		
HOMA-IR												
10	Randomized trials	Not serious	Not serious	Serious	Not serious	None	320	320	—	MD: 0.72 lower (1.07 lower to 0.38 lower)	⨁⨁⨁◯ Moderate	Critical
TG												
5	Randomized trials	Not serious	Not serious	Serious	Not serious	None	173	185	—	MD: 17.73 lower (29.55 lower to 5.9 lower)	⨁⨁⨁◯ Moderate	Critical
HDL												
5	Randomized trials	Not serious	Not serious	Serious	Not serious	None	183	185	—	MD: 0.37 lower (6.13 lower to 5.39 higher)	⨁⨁⨁◯ Moderate	Critical
LDL												
5	Randomized trials	Not serious	Not serious	Serious	Not serious	None	183	185	—	MD: 1.61 lower (6.35 lower to 3.13 higher)	⨁⨁⨁◯ Moderate	Critical
NO												
3	Randomized trials	Not serious	Not serious	Not serious	Not serious	None	82	80	—	MD: 0.47 lower (5.32 lower to 4.08 higher)	⨁⨁⨁⨁ High	Critical
hs-CRP												
5	Randomized trials	Not serious	Not serious	Not serious	Not serious	None	154	151	—	MD: 1.93 lower (2.3 lower to 1.56 lower)	⨁⨁⨁ High	Critical
FBG												
10	Randomized trials	Not serious	Not serious	Serious	Not serious	None	334	336	—	MD: 3.83 lower (6.49 lower to 1.18 lower)	⨁⨁⨁◯ Moderate	Critical
Insulin												
10	Randomized trials	Not serious	Not serious	Serious	Not serious	None	331	331	—	MD: 2.43 lower (3.37 lower to 1.48 lower)	⨁⨁⨁◯ Moderate	Critical

*CI*, confidence interval; *FBG*, fasting blood glucose; *HDL*, high-density lipoprotein; *HOMA-IR*, homeostasis model assessment insulin resistance; *hs-CRP*, C-reactive protein; *LDL*, low-density lipoprotein; *MD*, mean difference; *NO*, nitric oxide; *TG*, triglyceride.

### Synthesis of results

#### Primary outcome: homeostasis model assessment insulin resistance, fasting plasma glucose, and serum insulin

Of note, 10 studies reported HOMA-IR as a measure of metabolic control. A forest plot of the overall effect of probiotics and synbiotics on HOMA-IR among patients with GDM is shown in Figure 1. The overall pooled estimate of the 10 studies showed an MD of −0.72 (95% CI, −1.07 to −0.38) using a random-effects model. Moreover, probiotics and synbiotics were found to significantly reduce FBG levels, with an MD of −3.79 mg/dL (95% CI, −6.24 to −1.34; *P*=.00), but with a considerable heterogeneity (*I^2^*, 83%; *P*=.00). Meanwhile, insulin had a reduction of −2.43 mg/dL (95% CI, −3.37 to −1.48; *P*=.00) with a considerable heterogeneity (*I^2^*, 83%; *P*=.00). The *z* test results for the overall effects showed statistical significance (*P*=.00), suggesting that probiotics significantly reduced insulin resistance.

#### Secondary outcome: lipid level and inflammatory biomarker

##### Assessment of efficiency on lipid level

Of note, 5 studies provided data on the effects of probiotics on lipid profiles. The pooled data showed no significant difference in the effect of probiotics on reducing LDL levels (MD, −1.61 mg/dL (95% CI, −10.36 to −4.18; *P*=.5) with a considerable heterogeneity (*I^2^*, 71%; *P*=.00) and HDL levels (MD, −1.61 mg/dL (95% CI, −10.36 to −4.18; *P*=.5) with a considerable heterogeneity (*I^2^*, 96%; *P*=.00) (Figure 5 and 6). Conversely, significant difference was observed in TG (MD, −17.73 mg/dL; 95% CI, −29.55 to −5.9; *P*=.00) with a considerable heterogeneity (I^2^, 93%; *P*=.00) (Figure 6).

##### Assessment of efficiency on inflammatory biomarker

**Figure 8:**
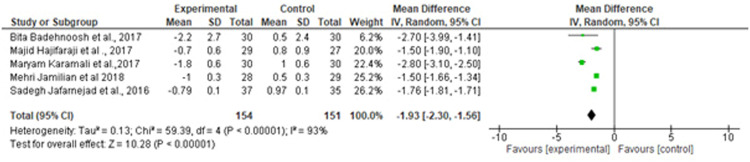
Forest plot mean difference of CRP *CI*, confidence interval; *CRP*, C-reactive protein; *IV*, inverse variance; *SD*, standard deviation.

**Figure 9:**
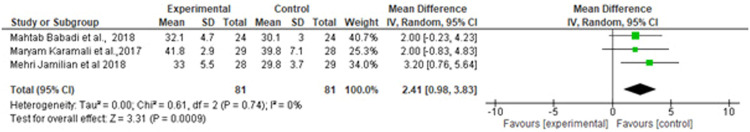
Forest plot mean difference of nitric oxide *CI*, confidence interval; *IV*, inverse variance; *SD*, standard deviation.

Of note, 4 studies involving 266 patients with 133 cases and 130 controls were identified. The combined data showed differences in CRP levels (MD, −1.93 dL; 95% CI, −2.3 to −1.56; *P*=.00) with a considerable heterogeneity (*I^2^*, 93%; *P*=.00) (Figure 8). NO levels did not show a statistically significant difference between the 2 groups (MD, −0.47; 95% CI, −5.02 to 4.08; *I^2^*, 79%; *P*=.84) (Figure 9).

Suastika. The role of probiotics and synbiotics on treatment of gestational diabetes. Am J Obstet Gynecol Glob Rep 2023.

Suastika. The role of probiotics and synbiotics on treatment of gestational diabetes. Am J Obstet Gynecol Glob Rep 2023.

### Meta-regression

Meta-regression analyses were performed to assess the effects of probiotics on the glycemic parameters in the GDM population. Our results revealed confounding variables, including the mean of maternal age (coefficient, 0.827; standard error [SE], −0.337; 95% CI, −0.337 to 0.552; *P*=.115), duration of follow-up (coefficient, 0.82; SE, −0.545; 95% CI, −0.545 to 2.185; *P*=.111), total probiotics dose (coefficient, 0.006; SE, 0.029; 95% CI, −0.016 to 0.029; *P*=.578), date of publication below 5 years (coefficient, −1.451; SE, 0.953; 95% CI, −3.318 to 0.417; *P*=.128), probiotic-only studies (coefficient, −0.021; SE, 0.059; 95% CI, −0.094 to 0.137; *P*=.304), study setting outside Iran (coefficient, 1.029; SE, 0.964; 95% CI, −0.862 to 2.919; *P*=.286), and risk of bias (coefficient, 0.469; SE, 0.897; 95% CI, −1.289 to 2.227; *P*=.601) (Table 4).

**Table 4:** Regression result effect of probiotic to HOMA-IR among GDM population

Covariate	Level	Studies	Coefficients	Lower bound	Upper bound	Standard error	*P* value
Intercept			−5.981	−13.417	1.454	3.794	.115
Mean of maternal age			0.087	−0.337	0.512	0.217	.687
Duration of intervention (mo)			0.820	−0.545	2.185	0.697	.239
Total dose (CFU)			0.006	−0.016	0.029	0.012	.578
Date of publication below 5 y	No	4	−1.451	−3.318	0.417	0.953	.128
	Yes	6					.128
Probiotic only	No	3	−0.714	−2.422	0.994	0.872	.413
	Yes	7					
Study place outside Iran	No	9	1.029	−0.862	2.919	0.964	.286
	Yes	1					
Risk of bias	Moderate	4	0.469	−1.289	2.227	0.897	.601
	Low	6					

*GDM*, gestational diabetes mellitus; *HOMA-IR*, homeostasis model assessment insulin resistance.

### Leave-one-out analysis

**Figure 10:**
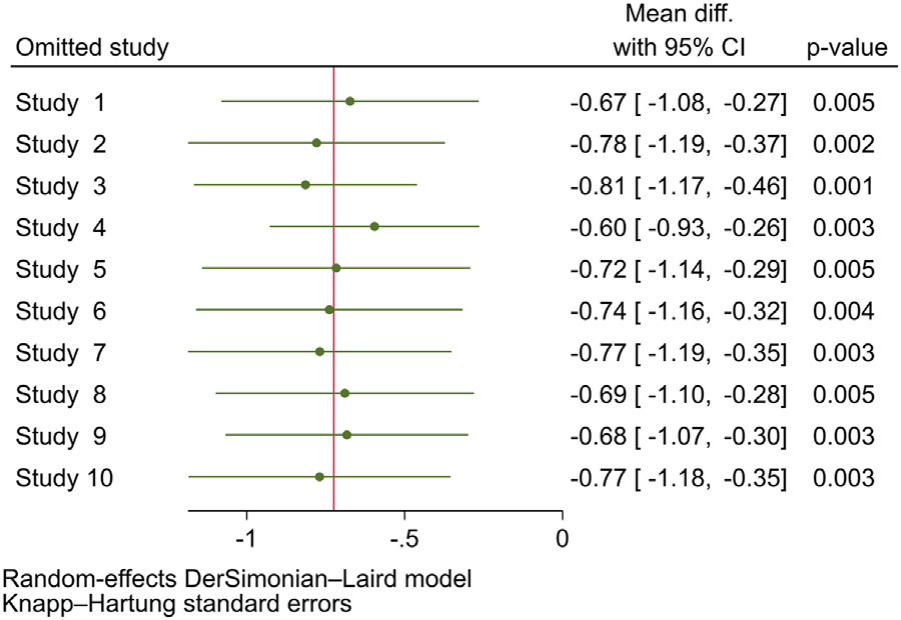
Leave-one-out analysis effect of probiotic to HOMA-IR among GDM population *CI*, confidence interval; *GDM*, gestational diabetes mellitus; *HOMA-IR*, homeostasis model assessment insulin resistance.

Sensitivity analyses of the effect of probiotics on HOMA-IR in the GDM population were conducted by excluding individual studies. The results indicated that the sensitivity exhibited robustness with an MD of −0.724 (95% CI, −1.120 to −0.329; *P*=.003) (Figure 10). Studies 3 and 4 seemed to have a relatively larger influence on the estimation of the overall effect size because of the roughly highest and lowest MDs compared with the influence of the others. In terms of clinical effect, a more pronounced effect was reported by Lindsay et al^22^ and Kamarali et al.^23^ However, the potential confounding factors in these 2 studies included single-strain probiotic administration and variations in physical pattern*.*

Suastika. The role of probiotics and synbiotics on treatment of gestational diabetes. Am J Obstet Gynecol Glob Rep 2023.

## Comment

### Main findings

Our meta-analysis included 13 trials involving 896 participants, with 150 in the intervention and 446 in the control group. We assessed the effects of probiotic and synbiotic supplementations for 4 to 8 weeks on glycemic control, insulin sensitivity, lipid profiles, and biomarkers of inflammation and oxidative stress among pregnant women with GDM. Our study concluded that probiotic and synbiotic supplementations significantly reduced insulin resistance (HOMA-IR and FSI), inflammatory markers (high-sensitivity CRP), oxidative stress (NO), and TGs; however, no significant difference was observed in the lipid profiles (LDL and HDL).

### Comparison with existing literature

We identified 6 systematic reviews that were similar to those in our study. The systematic reviews by Taylor et al^29^ and Pan et al.^30^ included 4 and 6 trials, respectively. Both studies assessed the effects of probiotics on GDM treatment. Our results for fasting plasma glucose (FPG) and HOMA-IR were similar to those of previous studies, with positive improvements. This positive result was also reported by Łagowska et al^31^ in 2020. The study analyzed 15 trials; however, 4 trials did not include patients with GDM. Lagowska et al^31^ reported that both probiotics and synbiotics have positive effects on glycemic control in patients with GDM but not in those without GDM. In contrast, a meta-analysis by Pan et al^32^ found that probiotic supplementation improved FPG, FPI, and HOMA-IR levels not only in patients with GDM but also among healthy pregnant women.

Recent studies have reported gut dysbiosis in women with GDM, with a gut microbiota composition resembling that of adults with T2DM.^33^^,^^34^ Kuang et al^35^ compared the gut microbiota composition between women with GDM and healthy pregnant women in the late second trimester of pregnancy and observed elevations in pathobionts, including *Parabacteroides distasonis, Klebsiella variicola*, and *Catenibacterium mitsuokai*. However, the expression levels of beneficial butyrate-producing bacteria, such as *Bifidobacterium* spp, *Eubacterium* spp, and *Methanobrevibacter smithii* were lower than those in healthy pregnant women. A study reported a reduction in the prevalence of GDM from 35% to 13% after probiotic supplementation with *Lactobacillus rhamnosus GG* and *Bifidobacterium animalis* subsp *Lactis BB-12*.^36^ Therefore, the gut microbiota is a potential marker of impaired glucose metabolism during pregnancy, and probiotic supplementation potentially has beneficial effects on glucose metabolism and, therefore, is a suggested GDM intervention.

Moreover, Taylor et al^29^ performed an analysis of the effect of probiotics on lipid profiles, especially LDL, and similar to our analysis, they found no statistically significant difference between the probiotic and control groups. Our study included 5 trials, whereas that by Taylor et al^29^ included 2 trials. A more recent systematic review by Zhang et al^37^ also found that probiotics may reduce TG levels, which is consistent with our meta-analysis. However, Zhou et al^38^ reported no significant difference in TG, HDL, and LDL levels between the probiotic and synbiotic and placebo groups.

During pregnancy, lipid metabolism undergoes physiological changes because of increased estrogen levels and insulin resistance in pregnant women, leading to decreased hepatic lipase activity and resulting in higher production of lipids in the liver.^39^^,^^40^ Women with GDM have higher levels of TGs, LDL, very LDL, and total cholesterol than those observed in normal pregnant women, with the largest differences observed in TG levels, whereas HDL levels were lower in women with GDM.^41^ Diet and gut microbiota may influence insulin resistance in women with GDM, which could be explained by the role of short-chain fatty acids (SCFAs) in energy metabolism. The gut microbiota ferments nondigestible fibers to produce SCFAs, such as acetate and butyrate. A high-fat, low-fiber diet in women with GDM may alter the normal gut microbiota composition, causing a decrease in butyrate-producing bacteria such as *Faecalibacterium* spp and *Firmicutes*, leading to low SCFA production. This decrease in SCFA in adipose tissue may lower the lipid storage capacity, resulting in elevated levels of free fatty acids in the circulation, which, in turn, may lead to increased lipid storage in the liver and muscle. Moreover, low levels of SCFAs may correlate with low-grade inflammation, because of the imbalance between anti- and proinflammatory cells.^42^ Bagarolli et al^43^ discovered that the administration of probiotics, *L rhamnosus, L acidophilus*, and *Bifidobacterium bifidum* in mice with diabetes mellitus prevented fat accumulation in the liver. Probiotics may regulate lipid metabolism by maintaining adipogenesis and fatty acid oxidation and suppressing lipolysis.^42^ However, further interventional studies are required to confirm these conflicting results.

### Strengths and limitations

This review was an updated meta-analysis that assessed the effect of probiotics and synbiotics in patients with GDM. However, this study had several limitations. First, our analysis revealed high heterogeneity because of the standard deviation (SD) in several studies on SE, which was converted into SD using the Cochrane calculator. The greatest drawback was the small number of RCTs that met the inclusion criteria in the final meta-analysis. Because of the limited number of studies, we could not compare the effects of probiotics and synbiotics separately. To analyze the heterogeneity, meta-regression analyses were performed to assess the effect of probiotics on HOMA-IR in the GDM population. Our results revealed confounding variables, including mean maternal age, duration of follow-up, total probiotic dose, date of publication below 5 years, probiotic-only studies, studies conducted outside Iran, and risk of bias not responsible for this heterogeneity. More RCTs are needed to provide detailed information and improve the review quality.

### Conclusions and implications

Probiotic and synbiotic supplementations reduce the risk of insulin resistance and improve glycemic control, blood lipid profiles, and inflammation in pregnant women with GDM. Probiotics are a possible option for GDM treatment; however, large-scale, well-designed RCTs with longer follow-up durations are required before they can be recommended to patients.

## Appendix Supplementary materials

Image, application 1
