# Supplementary material for: The role of probiotics and synbiotics on treatment of gestational diabetes: systematic review and meta-analysis
Source: AJOG Glob Rep. 2023 Oct 26;4(1):100285. doi: 10.1016/j.xagr.2023.100285 (PMC10844859; doi:10.1016/j.xagr.2023.100285)
Supplement: Supplementary file 1 [file mmc1.docx]

**Table 3.0** GRADE Approach

| **Certainty assessment** | | | | | | | **№ of patients** | | **Effect** | | **Certainty** | **Importance** |
| --- | --- | --- | --- | --- | --- | --- | --- | --- | --- | --- | --- | --- |
| **№ of studies** | **Study design** | **Risk of bias** | **Inconsistency** | **Indirectness** | **Imprecision** | **Other considerations** | **[intervention]** | **[comparison]** | **Relative (95% CI)** | **Absolute (95% CI)** |  |  |
| **HOMA-IR** | | | | | | | | | | | | |
| 10 | randomised trials | not serious | not serious | serious | not serious | none | 320 | 320 | - | MD **0.72 lower** (1.07 lower to 0.38 lower) | ⨁⨁⨁◯ Moderate | CRITICAL |
| **TG** | | | | | | | | | | | | |
| 5 | randomised trials | not serious | not serious | serious | not serious | none | 173 | 185 | - | MD **17.73 lower** (29.55 lower to 5.9 lower) | ⨁⨁⨁◯ Moderate | CRITICAL |
| **HDL** | | | | | | | | | | | | |
| 5 | randomised trials | not serious | not serious | serious | not serious | none | 183 | 185 | - | MD **0.37 lower** (6.13 lower to 5.39 higher) | ⨁⨁⨁◯ Moderate | CRITICAL |
| **LDL** | | | | | | | | | | | | |
| 5 | randomised trials | not serious | not serious | serious | not serious | none | 183 | 185 | - | MD **1.61 lower** (6.35 lower to 3.13 higher) | ⨁⨁⨁◯ Moderate | CRITICAL |
| **NO** | | | | | | | | | | | | |
| 3 | randomised trials | not serious | not serious | not serious | not serious | none | 82 | 80 | - | MD **0.47 lower** (5.32 lower to 4.08 higher) | ⨁⨁⨁⨁ High | CRITICAL |
| **hs-CRP** | | | | | | | | | | | | |
| 5 | randomised trials | not serious | not serious | not serious | not serious | none | 154 | 151 | - | MD **1.93 lower** (2.3 lower to 1.56 lower) | ⨁⨁⨁ High | CRITICAL |
| **FBG** | | | | | | | | | | | | |
| 10 | randomised trials | not serious | not serious | serious | not serious | none | 334 | 336 | - | MD **3.83 lower** (6.49 lower to 1.18 lower) | ⨁⨁⨁◯ Moderate | CRITICAL |
| **Insulin** | | | | | | | | | | | | |
| 10 | randomised trials | not serious | not serious | serious | not serious | none | 331 | 331 | - | MD **2.43 lower** (3.37 lower to 1.48 lower) | ⨁⨁⨁◯ Moderate | CRITICAL |

**Extended Analysis**

Of note, four studies provided data on the effect of probiotics on pregnancy outcomes. The pooled data showed no significant difference effect in macrosomia (risk difference of -0.08; 95% Confidence interval [CI], − 0.16 to − 0.00I^2^: 28.94%; p = 0.05); and caesarean section (risk difference of -0.13 ; 95% Confidence interval [CI], − 0.27 to − 0.01; I^2^: 42.78.% ; p = 0.06). but showed significant reduction in preterm delivery vs placebo (risk difference -0.49; 95% Confidence interval [CI], 0.54 to − 0.43; I^2^: 3.54%; p = 0.00)


Figure 11. Risk difference of Macrosomia between two groups

Figure 12. Risk difference of preterm delivery between two groups

Figure 13. Risk difference of caesarean section between two groups
